# Inspired Spine Smart Universal Resource Identifier (SURI): An Adaptive AI Framework for Transforming Multilingual Speech Into Structured Medical Reports

**DOI:** 10.7759/cureus.81243

**Published:** 2025-03-26

**Authors:** Jiawen Zhan, Dominic Moore, Yuanzhe Lu, Hamid Abbasi

**Affiliations:** 1 Machine Learning, Inspired Spine Health, Burnsville, USA; 2 Spine Surgery, Inspired Spine Health, Burnsville, USA; 3 Applied AI and Programming, Avicenna Technical University (ATU), Burnsville, USA; 4 Spine Surgery, Avicenna Technical University (ATU) and Inspired Spine Health, Burnsville, USA

**Keywords:** artificial intelligence in medicine, dictation software, language interpretation services, large language model(llm), multilingual, patient encounter

## Abstract

Medical documentation is a major part of delivering healthcare worldwide and is gaining more importance in developing countries as well. The global spread of multilingual communities in medical documentation poses unique challenges, particularly regarding maintaining accuracy and consistency across diverse languages. Inspired Spine Smart Universal Resource Identifier (SURI), an adaptive artificial intelligence (AI) framework, addresses these challenges by transforming multilingual speech into structured medical reports. Utilizing state-of-the-art automatic speech recognition (ASR) and natural language processing (NLP) technologies, SURI converts doctor-patient dialogues into detailed clinical documentation. This paper presents SURI’s development, focusing on its multilingual capabilities, effective report generation, and continuous improvement through real-time feedback. Our evaluation indicates a 60% reduction in documentation errors and a 70% decrease in time spent on medical reporting compared to traditional methods. SURI not only provides a practical solution to a pressing issue in healthcare but also sets a benchmark for integrating AI into medical communication workflows.

## Introduction

Background

Existing solutions in medical documentation have made strides toward improving efficiency but often fall short of addressing the core challenges of time consumption, cost, and error rates. Traditional electronic health record (EHR) systems, though widespread, require manual input, contributing to physician burnout [1]. Efforts to incorporate dictation tools and scribe services may prove useful but could be limited for some by cost and consistency issues, especially for providers whose native language is not English or who use languages other than English or major European languages. Recently, advancements in AI and machine learning have paved the way for automatic transcription and natural language processing (NLP)-driven tools. However, these tools are often constrained by limited linguistic capabilities and struggle with specialized medical terminology and utilization due to a lack of a solid framework that is compliant with the Health Insurance and Portability Accountability Act (HIPAA) [2].

SURI enters this landscape as a transformative solution. By integrating ASR and NLP with adaptive learning algorithms, SURI offers a new approach to documentation, one that is both multilingual and adaptive. SURI's open-source nature allows for fine-tuning based on medical-specific datasets, enabling it to handle complex dialogues with high accuracy across various languages.

The process of transforming multilingual speech into structured medical records relies on a combination of advanced technologies, which can be fine-tuned to meet specific requirements due to their open-source nature.

Automatic speech recognition (ASR) has evolved from early systems like Bell Labs’ Audrey in the 1950s [3] to modern deep learning-based models that achieve near-human transcription accuracy. Initially limited to recognizing a small vocabulary, ASR advanced through the introduction of hidden Markov models and statistical methods in the 1970s and 1980s [4], followed by the deep learning revolution in the 2010s, which dramatically reduced error rates and improved adaptability [5]. In medical settings, ASR faces challenges due to specialized terminology, diverse accents, and noisy environments, making accurate transcription difficult. However, recent advancements in open-source deep learning models have significantly improved ASR’s ability to handle these complexities [6]. The adaptability of these systems allows fine-tuning of medical-specific datasets, ensuring precise multilingual transcription while accommodating variations in speech and background noise. This flexibility is crucial in capturing spoken interactions between healthcare providers and patients accurately, aligning ASR output with the stringent requirements of medical documentation.

Natural language processing (NLP): Once the ASR system transcribes the speech, the next step is to convert the unstructured text into a structured format suitable for medical records. NLP has evolved from early rule-based language processing in the 1950s [7] to modern deep learning-driven approaches that power today’s advanced text understanding systems [8]. Initially focused on syntactic and rule-based parsing, NLP advanced through the development of statistical models in the 1980s and 1990s, allowing for more flexible and probabilistic language understanding [9]. In medical settings, NLP plays a crucial role in transforming unstructured ASR-generated text into structured formats for clinical documentation. This process involves extracting key medical entities such as diagnoses, treatment plans, and patient history using techniques like named entity recognition and relation extraction. Many NLP frameworks, such as cTAKES and BioBERT, are open-source and can be fine-tuned on domain-specific datasets to enhance accuracy and adherence to clinical documentation standards [10]. Advanced NLP methods, including syntactic parsing and context-aware text generation, further structure and refine medical transcripts, ensuring they are comprehensive and aligned with electronic health record requirements.

Adaptive learning algorithms: To ensure continuous improvement and adaptability, adaptive learning algorithms are integrated into the system. It has evolved from early artificial intelligence research, where pioneers demonstrated that machines could improve from experience rather than relying solely on predefined rules [11]. Over time, this concept led to modern continuous learning techniques that allow AI systems to refine their performance as they encounter new data [12]. In medical AI, adaptive learning plays a critical role by enabling systems to update their knowledge base dynamically, ensuring that recommendations and decisions align with evolving clinical standards. These algorithms incorporate feedback from healthcare professionals and integrate the latest medical research, allowing AI systems to continuously enhance their accuracy and effectiveness [13]. Additionally, the open-source nature of many adaptive learning frameworks facilitates collaborative refinements, where developers and clinicians can fine-tune models to keep pace with advancements in medical terminology and treatment protocols [14]. This self-learning capability is essential for reducing errors over time, improving overall system performance, and making AI-driven healthcare tools more responsive to the changing needs of medical professionals.

The challenge

Medical documentation remains a cornerstone of patient care, yet it is fraught with inefficiencies that strain both healthcare providers and institutions. A significant portion of this burden stems from the traditional methods of documentation, where physicians either personally record patient interactions or rely on clinical scribes. Both approaches are resource-intensive and contribute to systemic issues of time consumption, cost, and error rates.

Providers, on average, spend up to 44% of their workday on documentation [15]. This not only limits the number of patients a provider can see but also contributes to burnout, as clinicians extend their working hours to complete required documentation. Over time, this creates a ripple effect, reducing the overall quality of care delivered.

In many institutions, large dictation programs are relied upon to assist providers with documentation. These programs, such as DragonⓇ (Nuance, Burlington, Massachusetts, USA): a reference to a mythological creature that is large, cumbersome, and resource-intensive-demand a steep learning curve and significant time for review and correction, particularly for non-native English speakers [16]. Furthermore, the financial burden of implementing such automated dictation systems remains considerable for institutions.

In an attempt to mitigate these pressures, many healthcare institutions have turned to clinical scribes. While studies suggest that scribes can improve physician efficiency, the solution comes at a significant cost. The average annual expense for a scribe ranges from $47,000 to $50,000 [17-19], an investment that requires a physician to see at least 1.3 new patients daily to break even within a year (American Medical Association). Furthermore, the high turnover rate among scribes often results in a costly cycle of recruitment and retraining, diminishing the long-term cost-effectiveness of this solution.

Scribe usage also introduces variability in documentation quality. A scribe’s judgment can significantly affect the accuracy and completeness of medical records. Inconsistent or incomplete documentation can lead to medical errors, which account for 9.5% of deaths annually in the U.S. [20], illustrating the life-threatening consequences of imprecise record-keeping. The reliance on scribes, while alleviating some immediate workload, fails to address the core issues of accuracy and cost, leaving room for a more efficient and scalable solution.

## Materials and methods

Sample selection

This study utilized 6,258 audio recordings collected during real-world clinical interactions as part of pilot studies in standard clinical settings. Each recording was reviewed and verified by both the healthcare provider and a clinical assistant to ensure accuracy. Recordings were obtained from patients who provided informed consent for audio documentation during their clinical encounters or from provider summaries recorded after the patient visit.

The interactions occurred in typical clinical rooms, involving providers, patients, and occasionally, patient relatives. Patients and their relatives were not instructed to alter their behavior during recordings, ensuring naturalistic interactions. To improve the documentation process, providers adapted their behavior slightly by verbalizing physical examination findings and articulating all necessary details for medical records during the encounter.

The majority of the recordings were conducted in English, while 21 foreign languages were represented, including Arabic, Chinese, Dutch, English, French, German, Hindi (with seven distinct dialects), Italian, Japanese, Korean, Persian, Russian, Spanish, Thai, and Vietnamese, showcasing the multilingual capabilities of the system. This linguistic diversity allowed for an evaluation of SURI's performance across different languages and accents.

Testing environment

All recordings were obtained in real-world clinical settings, reflecting typical environmental conditions such as background noise, variable speech clarity, and spontaneous conversational flow. These pilot studies included both successful and challenging cases, ensuring that recordings with transcription errors were incorporated. This approach enabled the identification of SURI’s limitations and informed iterative improvements in the system’s performance.

Audio review and verification

Each recording underwent a thorough dual verification process to ensure transcription accuracy and support system improvement. Initially, healthcare providers and clinical assistants reviewed the transcription output generated by SURI, carefully identifying errors and inconsistencies in the documentation. These corrections were subsequently used to refine SURI’s adaptive learning algorithms, enabling the system to progressively improve its performance. This verification process not only ensured the accuracy of the transcriptions but also identified specific cases where SURI’s performance was suboptimal. These insights proved instrumental in guiding future updates to the model, addressing limitations, and enhancing its overall effectiveness in real-world clinical settings.

Statistical analysis methodology

A total of 6,321 audio recordings were collected during real-world clinical interactions for this study. We applied specific inclusion and exclusion criteria to ensure data quality and reliability. Inclusion criteria required that recordings were created for medical documentation purposes and had undergone review by both a healthcare provider and a clinical manager with comprehensive medical knowledge. Exclusion criteria eliminated recordings used in the testing phase and those without sufficient review.

Statistical analysis was conducted using a Python-based comparison script designed to evaluate differences between AI-generated transcriptions and finalized medical records. The methodology involved identifying and pairing corresponding 'clean' and 'unclean' medical documents based on filename patterns while ensuring the removal of non-essential content such as consent forms before proceeding with the comparison. The content discrepancy analysis utilized the difflib SequenceMatcher algorithm to assess the level of edits required. Modifications were categorized into three groups: minor additions, substantial edits, and deletions.

## Results

Statistical analysis results

After applying the inclusion and exclusion criteria, 2,786 recordings were selected for statistical evaluation. During the analysis, 117 recordings were excluded due to a 100% discrepancy between the AI-generated transcription (SURi) and the final medical record, resulting in 2,669 valid data points for the final analysis.

The modifications observed were categorized into three groups, where minor additions accounted for 12.5% of cases, substantial edits comprised 9.2%, and deletions constituted 8%. The mean modification rate was calculated at 26.95%, reflecting the proportion of AI-generated content that necessitated revision before finalization. The standard deviation was determined to be 22.43, indicating variability in the modifications attributed to differences in language, medical scenarios, and recording quality. Additionally, the variance was calculated as 503.12, underscoring the wide distribution in AI-generated documentation accuracy. A 95% confidence interval was established at (26.09%, and 27.80%), reinforcing the statistical reliability of these findings.

**Figure 1:**
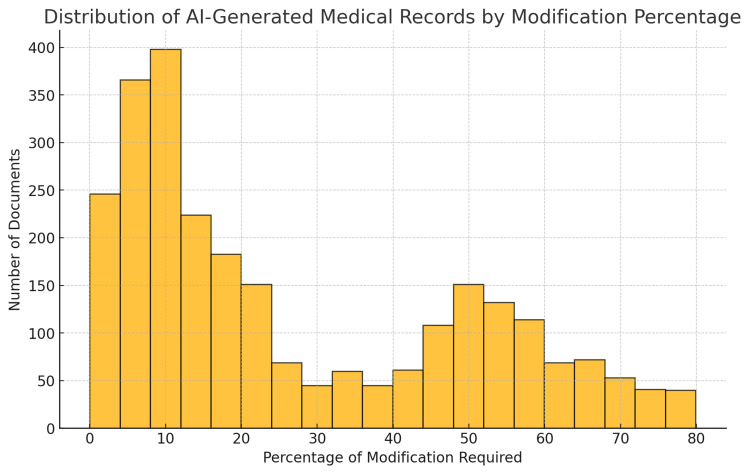
Distribution of all AI-generated medical records samples by modification percentage: This histogram categorizes documents based on the percentage of modifications required, grouped in 10% increments. It visually represents the number of documents that required different levels of changes, highlighting the frequency of minimal, moderate, and extensive modifications. Image Credits: Jiawen Zhan

These findings were visualized in Figure 1, which represents the distribution of AI-generated medical records based on the percentage of modifications required before finalization. The majority of records required minimal changes, with a peak around the 0-10% range, indicating high initial accuracy. However, some cases required more substantial modifications, particularly around the 50% mark, which warranted further investigation regarding potential causes.

By closely examining records with approximately 50% modification rates, it was found that additional standardized language related to standardized procedural documentation (bracing) and physician attestations was appended to the records. These additions were calculated as modifications due to errors, even though they were legitimate inclusions. However, the frequency and nature of these additions varied from patient to patient, which precluded systematic programmatic exclusion of these elements from the quantitative analysis. As a result, it is reasonable to infer that the actual modification rate is lower than the shown average.

Multilingual performance and error sources

The AI system processed medical transcriptions across 21 languages, with English accounting for the majority of cases, comprising 2,706 records. The next most frequently represented language group consisted of Hindi dialects with 21 transcriptions, followed by Chinese with 6 and Korean with 4. The remaining languages, including Arabic, Dutch, French, German, Italian, Japanese, Persian, Russian, Spanish, Thai, and Vietnamese, were represented by only one transcription each, largely collected during interactions with international fellows or visiting physicians. The limited number of recordings in these languages restricted the ability to generalize performance across multilingual settings.

Unlike traditional speech recognition benchmarks such as the Soniox and Whisper Speech Recognition Benchmarks (March 2023) [21], which evaluate Word Error Rate (WER) using predefined text comparisons, this study focused on real-world clinical encounters where speech is unscripted and lacks exact reference transcripts. Given the spontaneous nature of provider-patient interactions, direct WER measurement was not feasible. Instead, transcription accuracy was initially assessed through manual review, particularly in cases where the AI-generated record deviated significantly from the final medical documentation. This issue was first identified in the early stages of the study when physicians self-reported poor transcription performance in certain cases. Upon further investigation, a manual review of selected recordings confirmed that overlapping speech and background noise were the primary factors affecting transcription accuracy. In scenarios with multiple speakers, the ASR model tended to prioritize the most prominent voice, leading to the omission or misrepresentation of quieter voices, which contributed to significant errors in the final transcription.

As a temporary solution in later iterations of the study, physicians adapted their verbal documentation practices to improve transcription accuracy. When important information was conveyed during the overlapping speech, doctors intentionally repeated critical details to ensure the system captured them accurately. While the number of manually reviewed recordings was not systematically tracked, these observations played a crucial role in identifying speaker separation and noise resilience as key areas for future improvements in AI-driven transcription models.

Economic impact

The adoption of AI-driven transcription led to a significant reduction in documentation costs, with an 88.17% decrease in annual expenditures. Prior to AI implementation, medical documentation relied on five human scribes, each earning $20 per hour and working 40 hours per week, which resulted in an annual cost of approximately $208,000, excluding additional expenses associated with recruitment and training. With the transition to AI-based transcription, the total annual cost of documentation was reduced to $24,600, comprising $1,000 per month for server hosting, $1,000 per month for development and maintenance, and $50 per month for model hosting. This dramatic reduction in costs highlights the financial viability of AI-assisted medical documentation. As the system continues to improve and requires fewer manual corrections, it is expected that the costs associated with development and maintenance will decrease further, reinforcing the long-term economic sustainability of AI-integrated documentation systems in healthcare institutions. The cost-benefit analysis indicates an 88% reduction in documentation expenses, representing a substantial return on investment for healthcare institutions implementing similar AI-assisted documentation systems.

## Discussion

SURI: a solution

To address the challenges associated with traditional medical documentation methods, we introduce SURI (Inspired Spine’s Smart Universal Resource Identifier)-an adaptive AI framework specifically designed to transform multilingual speech into structured medical reports. SURI’s architecture is built on the integration of cutting-edge ASR and NLP technologies, allowing it to accurately transcribe and structure medical dialogues across multiple languages.

SURI's ASR component is tailored to meet the needs of diverse linguistic environments, ensuring that medical professionals can communicate with patients in their native languages without compromising the quality of the documentation. This multilingual capability is essential in global healthcare settings, where language barriers can lead to miscommunication and errors in patient records.

Once the ASR system transcribes the spoken dialogue, SURI's advanced NLP methods take over to convert the unstructured text into a coherent and standardized medical report. By leveraging NLP, SURI ensures that the generated reports are not only accurate but also contextually relevant, capturing the nuances of each patient interaction. 

A standout feature of SURI is its ability to learn and adapt over time. By incorporating feedback from users and integrating the latest medical advancements, SURI continuously refines its algorithms, enhancing its ability to generate high-quality medical documentation. This adaptive learning ensures that SURI remains a cutting-edge tool, capable of meeting the evolving demands of healthcare professionals and institutions.

**Figure 2:**
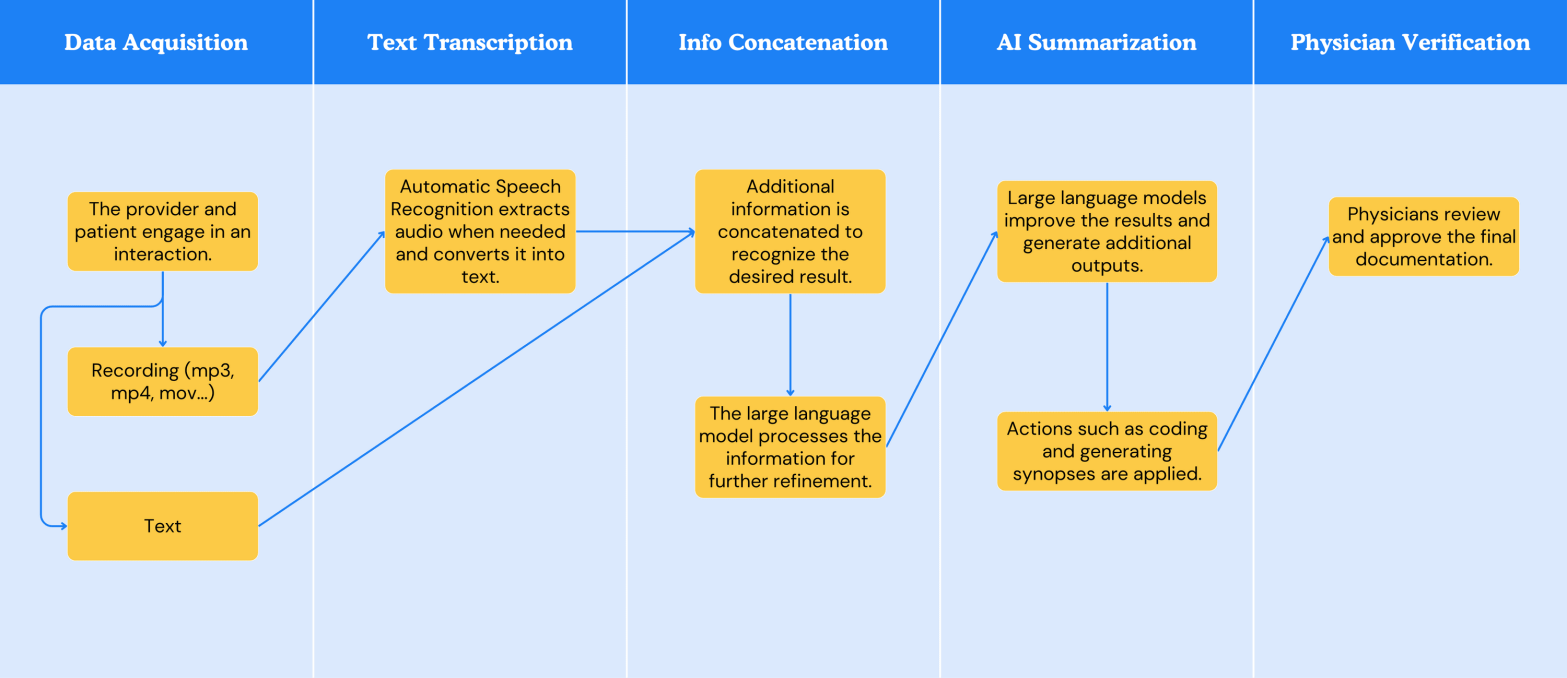
A sample workflow in text illustrating Inspired Spine Smart Universal Resource Identifier’s transcription and documentation process, showcasing the transformation of diverse input sources into a structured and organized output. Image Credits: Jiawen Zhan

**Figure 3:**
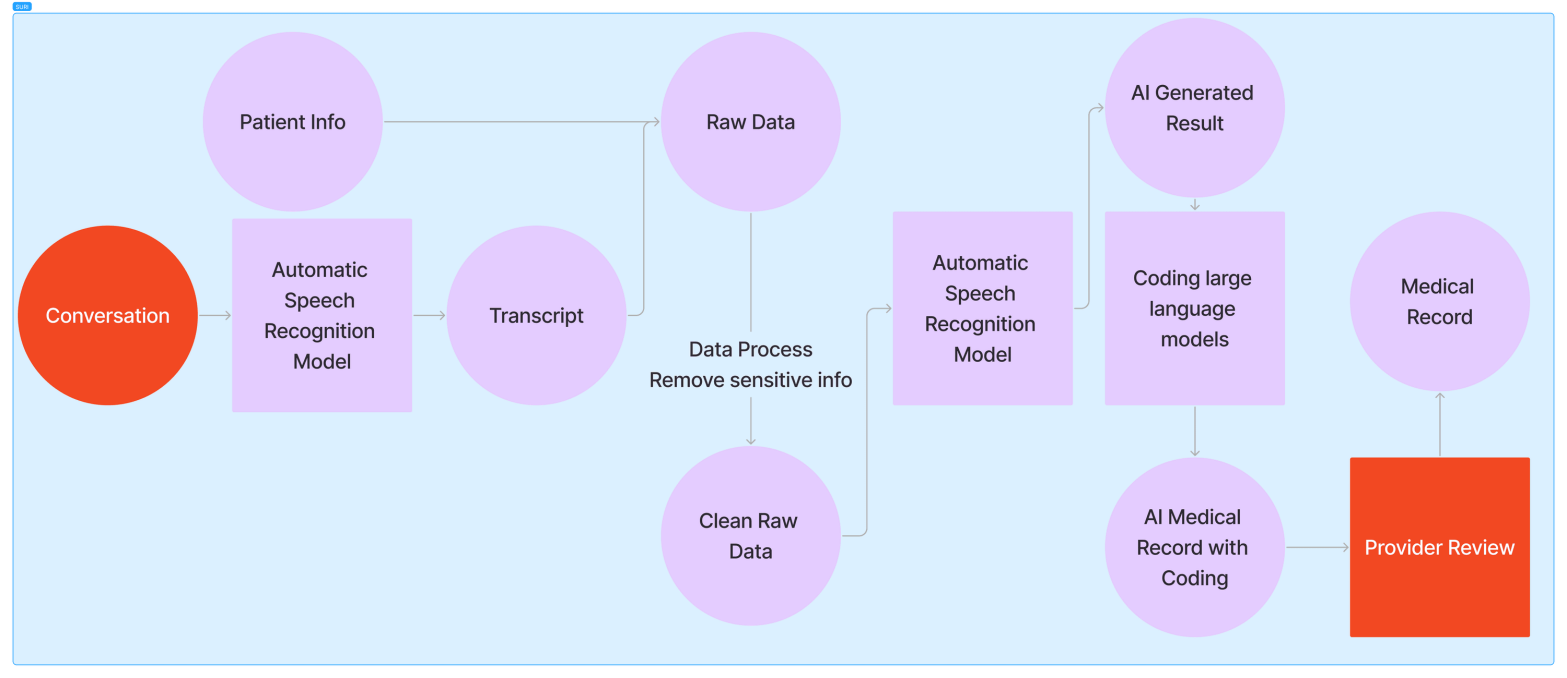
A sample workflow visually illustrating Inspired Spine Smart Universal Resource Identifier’s transcription and documentation process, showcasing the transformation of diverse input sources into a structured and organized output. Image Credits: Jiawen Zhan

By its design, NLP systems are not rigidly tailored to a single language. With appropriate adjustments, these systems can be adapted for use across different languages and even serve as effective platforms for translating local languages into English and vice versa. Advancements in cross-lingual learning and neural machine translation (NMT) have further improved this capability, making cross-language applications increasingly reliable and nuanced. The process is outlined in Figures 2, 3.

In summary, SURI offers a comprehensive solution to the challenges of medical documentation, streamlining the process, reducing costs, and improving accuracy. By integrating ASR, NLP, and adaptive learning, SURI aims to set a new standard for medical reporting, making it an invaluable asset in the healthcare industry.

Advances in automatic speech recognition for healthcare

ASR technology has advanced significantly, evolving from primitive voice recognition systems to sophisticated platforms capable of understanding complex medical terminologies across various languages. Amodei et al. (2016) demonstrated significant improvements in ASR performance using deep neural networks, achieving lower word error rates even in noisy environments, common in medical settings [22]. Researchers like Jaitly and Hinton have explored neural network acoustic models and transfer learning, enhancing ASR accuracy across languages-critical for a system like SURI that handles multilingual inputs [23].

AI in medical report generation

Recent advances in artificial intelligence, particularly in machine learning and NLP, have been pivotal in transitioning from manual to automated medical report generation. Studies by Zhang et al. on large language models (LLMs) like GPT-3 have shown the potential for generating detailed and contextually relevant medical reports from sparse or complex data [24]. These frameworks, trained on extensive datasets, can produce human-like text, crucial for accurate medical documentation.

Building upon these advancements, the rapid development of cutting-edge artificial intelligence models, both open-source and proprietary, has further expanded the potential of LLMs. These sophisticated systems have unlocked virtually limitless possibilities in information synthesis and report generation. By continuously improving their contextual understanding and output quality, modern LLMs are setting new benchmarks for generating precise, coherent, and highly relevant medical reports. This ongoing evolution ensures that the final reports become increasingly accurate and comprehensive, addressing the nuanced demands of medical documentation with remarkable efficiency.

Challenges and future directions

Implementing ASR and AI in medical settings presents challenges, particularly in noisy environments with varied speakers and specialized jargon. Ensuring the privacy and security of sensitive medical information is paramount. Future improvements should focus on adaptability and performance, leveraging continuous learning and feedback mechanisms. Researchers like Silver et al. suggest that reinforcement learning could dynamically enhance AI systems based on user interactions and feedback [25].

SURI's architecture and components

**Figure 4:**
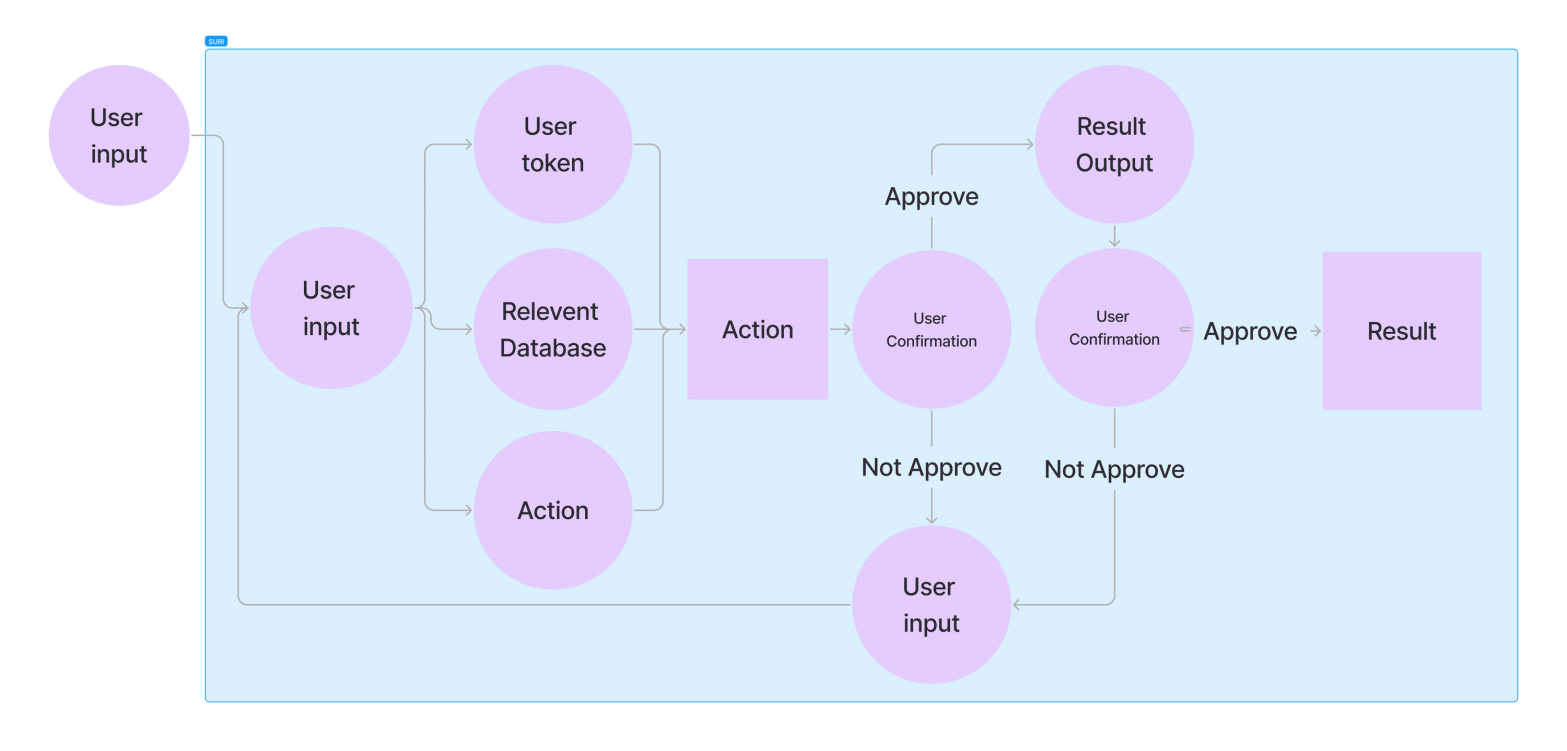
A sample workflow illustrating Inspired Spine Smart Universal Resource Identifier’s audio recognition function, demonstrating how natural language input is processed and transformed into actionable outputs executed by the system. Image Credits: Jiawen Zhan

SURI is a next-generation AI platform designed to improve the speed and quality of medical documentation. It leverages advanced technology to convert doctor-patient conversations into official medical records, considering the complexities of multilingual medical transcriptions. An example of its workflow is best illustrated in Figure 4.

Multilingual speech recognition module

In many regions of the world, healthcare providers and patients communicate in their native languages during consultations. However, formal medical documentation is often standardized in English, particularly in international settings. This global dynamic is also observed in countries like the United States, where healthcare providers and patients frequently speak different languages due to the diverse population. Providers may be multilingual, and technologies like teleconsultation have expanded the reach of cross-lingual healthcare services.

Given these complexities, SURI's multilingual speech recognition module plays a critical role in bridging the language gap. It not only transcribes conversations in multiple languages but also facilitates the transition of spoken language into standardized medical records, typically in English, ensuring that the documentation remains accessible and comprehensible to all healthcare professionals. This feature is vital for a system designed for universal use, enabling seamless integration across different linguistic and cultural contexts while supporting the needs of diverse patient populations.

Transformer-based large language model (LLM)

SURI's language processing capabilities are powered by a fine-tuned version of LLaMA 3.5, chosen for its adaptability and precision in handling complex clinical language across multilingual settings. This model is fine-tuned using real historical patient interactions and corresponding medical records, with all patient identifiers omitted to ensure confidentiality. By training on real-world clinical data, LLaMA 3.5 captures the subtleties of patient-provider interactions, translating them into clear, structured medical documentation.

In addition to dataset-specific tuning, the model’s weights were carefully adjusted to balance creativity and tone, ensuring that SURI’s output maintains a professional, clinical standard while accurately reflecting patient conversations. Prompt engineering was applied to refine response precision, tailoring the model’s responses to the specific requirements of structured medical documentation.

**Figure 5:**
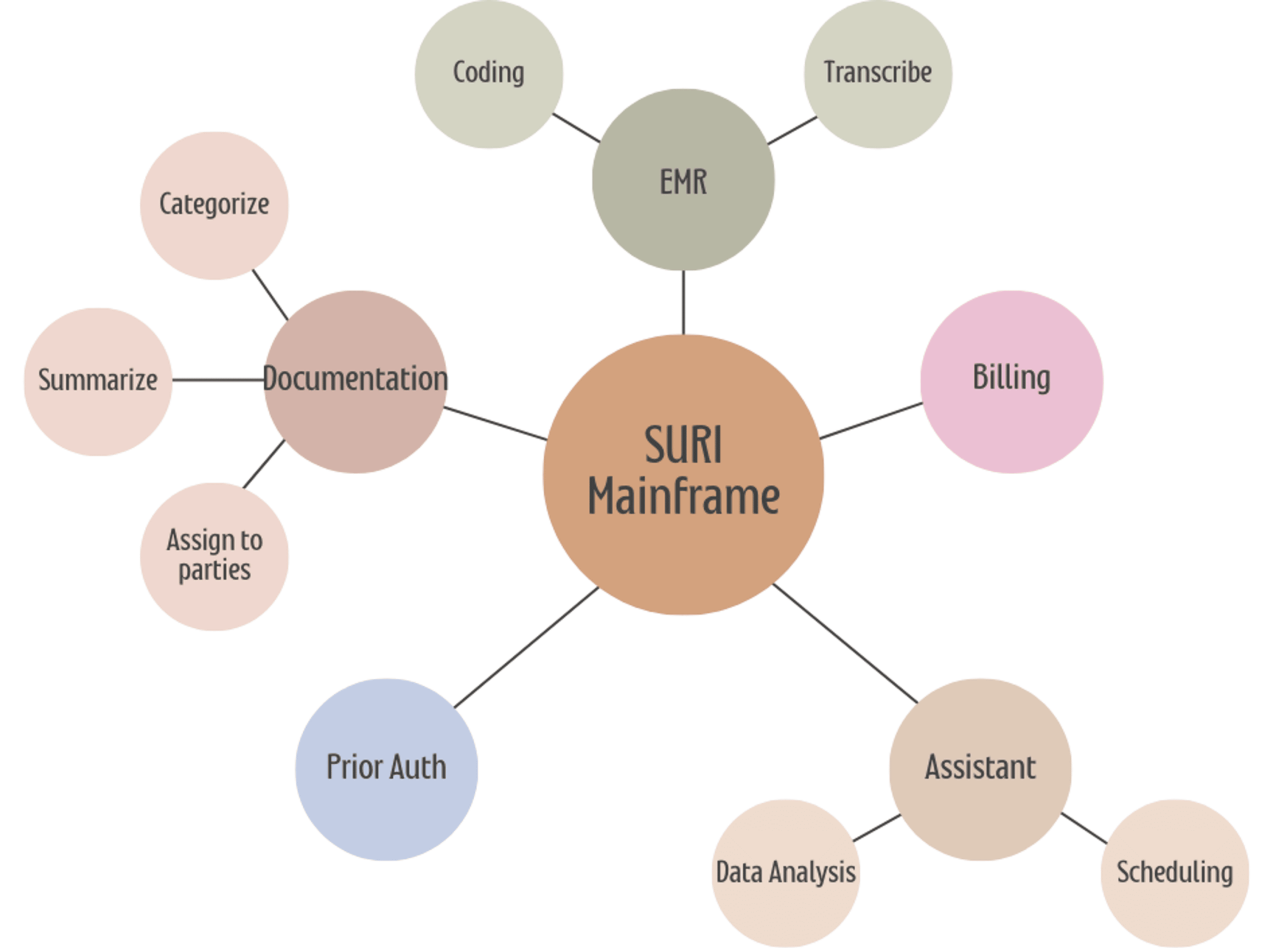
Overview of Inspired Spine Smart Universal Resource Identifier's mainframe structure and its interconnected functions. The diagram illustrates the core components of Inspired Spine Smart Universal Resource Identifier, including documentation, transcription, billing, scheduling, data analysis, prior authorization, and coding, along with their specific tasks such as categorization, summarization, and assignment. Each function interacts seamlessly with the mainframe to support efficient and streamlined medical operations. Image Credits: Jiawen Zhan

The fine-tuning process also incorporates advanced techniques, including supervised learning and reinforcement learning with human feedback (RLHF). These techniques align the model’s outputs with human preferences for accuracy and clarity, ensuring that the generated documentation adheres to clinical standards while capturing the intricacies of patient-provider dialogues. This level of refinement ensures that the documentation is not only useful for immediate clinical care but also suitable for long-term medical records, supporting continuity and quality in healthcare in a variety of ways, as shown in Figure 5.

SURI’s transformer architecture, enhanced with these advanced methodologies, enables the model to handle long sequences of text, generating coherent and organized reports that encompass various sections such as diagnoses, treatment plans, patient histories, and follow-up instructions. With its highly specialized tuning, SURI consistently produces accurate, contextually relevant documentation, offering healthcare professionals a reliable, time-efficient tool for capturing essential details from clinical interactions.

Technology stack for development and deployment

SURI's development centers on a powerful C#-based architecture, utilized as the primary language for both the front and backend. This choice enables highly structured data processing, seamless integration with healthcare systems, and reliable performance, supporting the complex requirements of real-time medical documentation. JavaScript is incorporated only for specific supporting functions, enhancing the user experience with additional interactive elements.

For data storage and database management, SURI relies on Amazon Web Services (AWS, Seattle, USA), which provides a secure, scalable, and HIPAA-compliant environment essential for handling sensitive medical data. AWS offers encryption, access control, and flexible scaling, which allow SURI to efficiently manage the high volumes of data typical in healthcare documentation. By using AWS, SURI ensures rapid data retrieval and processing, enabling real-time learning and adaptation to user feedback.

Together, these technologies support SURI's efficient, scalable deployment across various healthcare settings, making it adaptable to the documentation needs of global, multilingual healthcare environments.

Adaptive learning capabilities

SURI’s adaptive learning capabilities enable it to continually improve its performance over time by fine-tuning based on real-world feedback. The system not only learns from user input but also tracks changes made to the final medical reports. Each adjustment and refinement are logged, and these modifications are compiled into new training datasets. By incorporating these iterative changes, the model automatically fine-tunes itself, progressively aligning its outputs with clinical expectations.

One of the key aspects of SURI’s learning is its ability to document and analyze the revisions made to its generated reports. This process allows the model to identify patterns in the adjustments and understand which areas are most critical. By recognizing these priorities, SURI can intelligently adapt its outputs to the specific needs of both individual users and healthcare organizations, thereby refining the quality of documentation with each interaction.

Continuous learning 

It constantly compares the AI-generated version to changes done by the user, and after patterns of corrections are recognized, these are implemented automatically for the same user. If multiple users across one institution make the same changes, these learning points are institutionalized, and if across multiple institutions the same changes are implemented, they become global learning points for the AI system.

It gradually reduces the need for human intervention in the final documentation process. Over time, SURI becomes more accurate and efficient, consistently delivering medical records that require fewer edits or corrections. With each iteration, the platform provides more reliable and polished reports, eventually enabling healthcare providers to trust the output with minimal manual adjustments, thereby saving time and improving overall documentation workflow.

In addition to pattern recognition and correction, SURI incorporates a keyword change identification mechanism to further enhance its self-learning capabilities. This feature smartly tracks and analyzes changes to key terms in the medical documentation, assessing their criticality based on context and frequency of edits. For instance, if a particular term is consistently replaced or adjusted by users, SURI flags it as a potential improvement point and evaluates its relevance within similar contexts. Over time, SURI builds a comprehensive library of commonly misused or ambiguous keywords, categorizing them by their criticality. This library is then shared across users, allowing individual providers to benefit from the collective insights of the larger user base. By leveraging this collaborative intelligence, SURI ensures that its output aligns more closely with the preferred terminology and standards of medical institutions, fostering a more accurate and efficient documentation process.

Personalized medical record tailoring

Through this dynamic method of training, SURI ensures that medical records are eventually tailored to match the unique habits and preferences of each individual user. Rather than applying a single model uniformly to all users, SURI customizes its outputs based on the specific tendencies of each healthcare provider, organization, or practice. This individualized approach guarantees that the system evolves to meet the precise documentation needs of each user, making the medical records as personalized as the care provided. As SURI continues to learn from the user’s specific style and practices, it not only improves efficiency but also enhances the overall satisfaction and trust in the documentation process.

Automated coding with SURI

SURI’s automated coding process combines hard-coded algorithms with the advanced capabilities of LLMs to ensure precise and efficient medical record coding. The hard-coded components handle direct and unambiguous matches for standardized codes, such as ICD-10 diagnosis codes and CPT codes for operations and treatments. Meanwhile, the LLMs are employed to interpret abstract or ambiguous diagnoses, leveraging their contextual understanding to assign the most accurate codes when straightforward matching is insufficient.

Much like SURI’s self-improving mechanism for medical records, its coding functionality continuously learns from user corrections. When users adjust or refine the assigned codes, SURI identifies patterns in these modifications, improving its coding accuracy over time. This iterative learning ensures that the system becomes increasingly adept at handling complex and nuanced cases, ultimately reducing the need for manual intervention. By integrating user feedback and continuously updating its processes, SURI delivers a robust, evolving solution for automated medical coding that aligns with evolving medical standards and practices.

Implementation

The implementation of SURI involves a careful integration of advanced technologies to ensure seamless functionality in medical environments while prioritizing data security, user experience, and future adaptability. Designed to complement existing healthcare workflows, SURI integrates with various healthcare information systems such as Electronic Health Records (EHRs), hospital management systems, and diagnostic tools. This compatibility is essential for accessing and processing audio data from clinical interactions and embedding the resulting structured reports back into patient records. SURI captures audio inputs through standardized APIs that connect to various recording devices used across medical facilities. Once transcriptions are processed, the structured reports are securely delivered back into the EHRs, ensuring data integrity and confidentiality throughout the process. Special attention is given to making SURI’s software components compatible with diverse healthcare IT infrastructures, including both modern and legacy systems, achieved through adaptable middleware capable of interfacing with varied platforms.

Data handling is a critical aspect of SURI’s implementation, given the sensitivity of medical information. The system acquires audio data directly from clinical interactions while employing automated mechanisms to remove personally identifiable information before processing, thus safeguarding patient confidentiality. All data processing, including transcription and report generation, occurs on secure servers utilizing encrypted storage and transfer protocols to protect against unauthorized access and potential breaches. This secure handling ensures compliance with healthcare data standards and regulations while maintaining the integrity of the processed information.

SURI’s user interface (UI) is designed to accommodate the varying technological proficiency levels of medical staff. The intuitive design prioritizes simplicity, featuring a clean layout with clear labeling and straightforward navigation to minimize cognitive load. By eliminating unnecessary complexity, SURI enables healthcare professionals to quickly adapt to the system and focus on patient care rather than operational challenges. An innovative feature of the interface includes an integrated guidance module where users can simply ask SURI how to perform specific tasks, and the system provides step-by-step assistance. This functionality not only improves user experience but also fosters greater adoption across diverse user groups.

To support continuous improvement, the interface incorporates real-time feedback mechanisms, allowing users to report issues or suggest enhancements directly within the system. These user-driven insights feed into SURI’s adaptive learning capabilities, enabling it to refine its outputs and improve accuracy and efficiency over time. The integration of feedback tools fosters a collaborative relationship between users and the system, ensuring that SURI evolves in line with real-world demands.

Looking to the future, SURI’s development roadmap includes the integration of advanced voice control and natural language processing capabilities, enabling entirely hands-free interaction. This functionality will allow users to manage medical records, dictate commands, and query the system in natural language, significantly reducing the learning curve and enhancing usability. Beyond simplifying documentation, SURI is envisioned as a multifunctional assistant capable of advanced data analytics and research support. By searching large datasets, generating insights, and interacting with users in a conversational manner, SURI will empower healthcare professionals to make data-driven decisions efficiently. The fusion of voice-controlled interaction and intelligent analytics aims to position SURI as an indispensable tool for improving clinical workflows, decision-making, and overall healthcare efficiency.

Evaluation

The evaluation of SURI is conducted through a multidimensional framework that rigorously examines both its technical performance and its acceptance within clinical environments. This comprehensive approach ensures that the system not only meets technical benchmarks but also aligns with the practical needs and expectations of its users.

SURI’s capabilities are assessed using well-defined performance metrics. The accuracy of speech recognition, a cornerstone of the system, is measured by the Word Error Rate (WER), where lower values indicate superior transcription accuracy. The quality of medical reports generated by SURI is evaluated through precision, recall, and F1-score, metrics that determine the relevance and comprehensiveness of the captured medical information. Additionally, the system’s capacity for adaptation and learning is tracked by observing reductions in error rates over time and its ability to integrate new terminologies or respond effectively to user feedback. From an economic perspective, SURI’s cost-saving potential is highlighted by comparing traditional scribe service expenditures with the operational costs of deploying the AI system, demonstrating its financial efficiency.

To ensure robust evaluation, SURI undergoes a systematic testing methodology. Initially, the system is subjected to controlled environment testing, where predefined audio samples and medical scenarios are used to fine-tune its performance in a predictable setting. This phase minimizes variables and allows for precise calibration. Once optimized, SURI progresses to pilot clinical trials, where it is deployed in real-world medical scenarios. This critical phase assesses the system’s interaction with existing healthcare IT systems, its adaptability to dynamic clinical workflows, and its usability by medical staff. Finally, a longitudinal study tracks SURI’s performance over an extended period, measuring its ability to learn from feedback, integrate new medical knowledge, and maintain consistent reliability.

User feedback plays a central role in evaluating SURI’s effectiveness, offering insights into its practical impact on clinical workflows. Regular surveys and interviews with medical staff provide qualitative data on user satisfaction, ease of use, and the perceived utility of the system. These insights are complemented by usability testing sessions, where users interact with SURI under observation, allowing evaluators to identify pain points and gather actionable suggestions for improvement. This user-centric approach ensures that the system evolves in response to real-world needs and expectations.

The evaluation process also incorporates rigorous data analysis and reporting to provide a comprehensive overview of SURI’s effectiveness. Quantitative data analysis involves the application of statistical methods to evaluate performance metrics and user activity logs, offering a clear picture of SURI’s efficiency, accuracy, and reliability. Meanwhile, qualitative assessments focus on extracting valuable themes and insights from user feedback, interview transcripts, and open-ended survey responses, providing context and depth to the evaluation.

By combining technical metrics, structured testing, and user-driven insights, the evaluation of SURI ensures a holistic understanding of its performance, reliability, and impact. This approach not only validates SURI’s current capabilities but also informs future enhancements, ensuring the system continues to meet the evolving demands of the healthcare environment.

Discussion

The implementation of SURI has demonstrated significant improvements in both the accuracy and efficiency of medical documentation, as shown by Figure 1, where the majority of records required minimal changes around less than 10%. By leveraging cutting-edge AI technologies, SURI’s multilingual ASR and NLP framework has successfully automated the process of converting diverse speech inputs into structured medical reports. Its ability to handle multilingual data, particularly in complex and noisy medical environments, positions it as a powerful tool for improving documentation quality and reducing errors.

In our randomized field study using a combined ASR and LLM system for medical documentation, we found that the auto-generated clinical notes still required a significant percentage of modifications by clinicians to ensure accuracy and completeness. This percentage-of-modification metric provides a practical measure of real-world usability, capturing the editing effort needed to address disfluencies and extraneous patient speech - factors often overlooked by traditional metrics like WER. Unlike prior ASR studies that primarily reported WER in controlled or simulated environments [26], our research accounts for the unpredictable nature of live patient-clinician conversations, which introduce random, tangential content that complicates direct transcription. To mitigate these issues, we integrated an LLM to filter out irrelevant details and correct transcription errors [27]. This approach builds on decades of ASR advancements that are grounded in foundational work by Baker (1975) on trainable speech recognition grammars [3].

In a comparable study, Artificial Intelligence Scribe and Large Language Model Technology in Healthcare Documentation: Advantages, Limitations, and Recommendations by Mess et al. (2024) [28], the authors explore similar objectives of integrating ASR and LLMs into healthcare documentation, focusing on the improvements to efficiency and patient-centered care. 

A standout benefit of SURI is its adaptive learning capability, which allows the system to refine its outputs continuously by incorporating user feedback and adapting to evolving medical terminologies. This self-learning feature ensures that SURI stays aligned with clinical best practices while catering to individual user preferences. Over time, this adaptability minimizes the need for manual corrections, further improving efficiency. However, a key challenge lies in balancing adaptability with strict data privacy protocols. Ensuring that the system's learning mechanisms do not compromise patient confidentiality will remain a critical focus as the system evolves.

Despite its strengths, user feedback has pointed to areas for improvement, particularly in the user interface (UI). While the system excels at streamlining documentation workflows, enhancements are needed to improve the ease of making corrections and navigating through the interface. Addressing these usability challenges will be key to increasing user adoption and maximizing the system’s potential benefits. Future iterations of SURI should prioritize refining the UI to ensure an intuitive and seamless user experience.

The economic benefits of SURI are undeniable. Its ability to replace traditional scribe services has resulted in a significant reduction in operational costs, demonstrating the financial advantages of integrating AI-driven automation into healthcare workflows. As the system continues to scale, these savings are expected to grow, solidifying SURI's position as a financially sound investment for healthcare institutions. Furthermore, its low operational costs, combined with its scalability, make it a sustainable and cost-effective solution for modern healthcare environments.

In summary, SURI represents a transformative approach to medical documentation, balancing technical precision, economic efficiency, and adaptability to meet the evolving needs of healthcare providers. By addressing its current limitations and building on its strengths, SURI has the potential to set a new standard for AI-driven documentation systems in the healthcare industry.

Limitations

While SURI demonstrates remarkable potential as a multilingual, adaptive AI framework for medical documentation, several limitations must be addressed to ensure broader adoption and optimal performance. One of the primary challenges lies in maintaining accuracy in noisy environments. Despite leveraging advanced automatic speech recognition (ASR) technology, SURI’s performance can decline significantly in settings with high background noise, overlapping conversations, or unclear speech. Such scenarios are common in busy clinical environments or during telehealth consultations, potentially impacting the system's usability.

Language and accent variability present another challenge. While SURI supports multiple languages, its effectiveness can vary depending on the rarity of the language or the prominence of regional accents. This variability could reduce inclusivity, particularly in linguistically diverse or underserved communities. Additionally, SURI relies heavily on the quality of input data and feedback for its adaptive learning capabilities. Inconsistent or incomplete feedback from users can slow the system’s learning curve, limiting its ability to adapt to new medical terminologies or best practices.

A potential concern with SURI’s automation capabilities is the risk of over-reliance by users. The convenience of automated documentation may inadvertently reduce users’ diligence in cross-checking for errors or omissions, which could compromise the quality of medical records in critical scenarios.

Future work

To address these limitations and enhance its capabilities, the future development of SURI focuses on improving performance, adaptability, and integration with evolving healthcare technologies. One priority is fine-tuning the system to better handle diverse accents and reduce noise-related errors. This involves collecting a broader range of voice samples and refining the system's algorithms to enhance recognition accuracy in challenging environments. Noise reduction improvements, particularly at the start of recordings, aim to prevent misinterpretation or hallucinations, where the model may default to the wrong language or make inaccurate assumptions due to initial noise interference.

SURI’s future scope also includes integration with advanced medical sensors and cameras. Moving beyond wearables like smartwatches, these tools could capture patient movements, physical observations, and other non-verbal cues during consultations. By incorporating image recognition and video reasoning technologies, SURI could enrich patient records with automated visual data interpretation, reducing the need for providers to manually describe their observations. This integration could significantly alleviate the documentation burden while providing a more holistic representation of patient interactions.

Further advancements in adaptive learning and predictive modeling will allow SURI to keep pace with medical innovations, evolving terminologies, and emerging healthcare challenges. By anticipating changes in medical language and adapting to real-time trends, SURI can remain a forward-thinking tool capable of addressing new clinical demands and diseases.

A key limitation identified in our observation is the tendency for transcription errors when multiple speakers overlap or when one speaker’s volume overshadows others. While this issue has thus far been noted qualitatively, future iterations of SURI will include systematic tracking of these overlap-related discrepancies to gather quantitative insights. By capturing precise metrics on how often such errors occur, we can better measure improvements over time and refine SURI’s speaker separation algorithms for more accurate transcriptions in multi-speaker environments.

The rapidly evolving AI industry also provides opportunities to enhance SURI’s capabilities. By incorporating cutting-edge developments in natural language processing, image recognition, and predictive analytics, SURI’s technical precision and user experience can be continually refined. Maintaining an open approach to adopting these advancements ensures SURI remains at the forefront of AI innovation in healthcare.

Finally, with the growing prominence of telehealth, SURI aims to seamlessly integrate into virtual healthcare platforms. By providing real-time transcription and report generation during remote consultations, SURI can uphold high documentation standards while allowing providers to focus more on patient care. This telehealth integration will enhance the quality of remote healthcare services, making SURI a vital tool for both in-person and virtual medical practices.

## Conclusions

SURI represents a major advancement in medical documentation, leveraging AI to streamline record-keeping for healthcare providers. By utilizing advanced ASR and NLP technologies, it accurately converts multilingual medical conversations into structured records, reducing the time and effort required for documentation. This automation not only lowers costs and enhances accuracy but also allows professionals to focus more on patient care. With adaptive learning capabilities, SURI continuously improves by integrating new medical terminologies and real-world feedback. Its potential extends beyond documentation, positioning it as a future research assistant and analytics tool for deeper insights into patient care. As it evolves, SURI will continue to enhance medical workflows, reduce administrative burdens, and improve healthcare efficiency.
